# Differential expression of prolyl hydroxylase 1 in patients with ulcerative colitis versus patients with Crohn’s disease/infectious colitis and healthy controls

**DOI:** 10.1186/1476-9255-10-36

**Published:** 2013-11-20

**Authors:** Sophie Van Welden, Debby Laukens, Liesbeth Ferdinande, Martine De Vos, Pieter Hindryckx

**Affiliations:** 1 Department of Gastroenterology, Ghent University Hospital, De Pintelaan 185, 3K12-IE, Ghent B-9000, Belgium; 2 Department of Pathology, Ghent University, Ghent, Belgium

**Keywords:** Prolyl hydroxylases, Crohn's disease, Ulcerative colitis, Infectious colitis

## Abstract

**Background:**

Inhibition of prolyl hydroxylases (PHDs) leads to the induction of a transcriptional program that, in the gut, promotes intestinal epithelial cell survival. PHD inhibitors have recently been suggested as a promising alternative treatment for inflammatory bowel disease (IBD). In this study, we explored the colonic mucosal expression of the different PHD-isoforms (PHD1, 2 and 3) in order to identify the key isoform(s) involved in the pathogenesis of IBD.

**Methods:**

The mRNA expression of inflammatory cytokines (IL-8 and TNF-α), an apoptosis marker (caspase 3) and PHD1, 2 and 3 was analysed in biopsies of IBD patients (UC and CD), patients with infectious colitis and healthy controls using qRT-PCR. PHD protein levels were evaluated using western blot. Cellular localization of PHD 1, 2 and 3 was determined by immunohistochemistry.

**Results:**

PHD1 was significantly up-regulated in IBD patients, both at the mRNA (UC: p < 0.0001 and CD: p < 0.05) and at the protein level (UC: p < 0.05 and CD: p < 0.05), and showed a very good correlation with the expression of the inflammatory cytokines IL-8 and TNF-α and the apoptosis marker caspase 3. Colonic mucosal PHD2 mRNA and protein expressions were not altered in IBD. PHD3 expression was increased in inflamed biopsies from UC patients (p < 0.0001), but only at the mRNA level. PHD1 and PHD2 expression was found both in the colonic lamina propria and the epithelium while PHD3 was mainly located in the endothelium of blood vessels.

**Conclusions:**

In this exploratory expression analysis, PHD1 comes forward as the primary therapeutic target for UC and, to a lesser extent, for (colonic) CD.

## Findings

### Background

Prolyl hydroxylase domain-containing proteins (PHDs) are oxygen sensing enzymes that, under normoxic conditions, hydroxylate the hypoxia-inducible factor 1 alpha subunit (HIF-1α), leading to its proteasomal degradation. During hypoxia, the PHDs are inhibited, leading to the formation of the active transcription factor HIF-1, which induces the expression of several cell survival genes (i.e. the hypoxic adaptive response) [1]. Several groups have proposed prolyl hydroxylase (PHD) inhibition as a promising novel strategy in the treatment of inflammatory bowel disease (IBD) [2-4].

To identify the key PHD isoforms (PHD1-3) involved in the pathogenesis of IBD, we explored their colonic mucosal expressions in endoscopically derived colonic mucosal biopsies from healthy controls and patients with Crohn's disease (CD), ulcerative colitis (UC) and infectious colitis.

## Methods

### Study populations and samples

Colonic mucosal biopsies were taken from endoscopically inflamed areas of 19 Crohn’s disease (CD) patients and 10 ulcerative colitis (UC) patients with active disease, and from completely healed mucosa of 16 CD patients and 5 UC patients in remission. Samples of 20 healthy controls (HC) and inflamed regions of 9 patients with infectious colitis were included as controls. Patients were diagnosed with infectious colitis based on histological findings (5 out of 9) or positive stool sample cultures (4 out of 9). The patients with infectious colitis were not known with IBD. Biopsies were stored immediately after removal in -80°C. IBD patients were either free of medication use or used 5-aminosalicylates in monotherapy. This study was approved by the ethical committee of the University Hospital of Ghent (permit number EC UZG 2004/242) and all participants gave their written informed consent. Patient characteristics are summarized in Table 1.

**Table 1:** Patient characteristics

***Group***	***Healthy controls***	***UC inflamed***	***UC remission***	***CD inflamed***	***CD remission***	***Infectious colitis***
N (Biopsies)	20	16	5	19	10	9
Gender (male/female)	5/15	8/8	4/1	8/11	4/6	6/3
Age, years (mean)	49	38	50	32	49	36
Age, years (range)	12-73	14-58	26-70	11-54	28-73	17-58
Age at diagnosis						
A1/A2/A3		1/9/6	0/4/1	4/11/4	0/6/4	
Max location of disease						
L1/L2/L3/L3 + L4/L4				0/7/9/3/0	3/2/4/0/1	
E1/E2/E3		3/10/3	0/4/1			
Max disease behaviour						
B1/B2/B3				12/3/4	4/3/3	
				(9^P^)	(4^P^)	
Medication						
No	20	5	3	13	8	9
5-aminosalicylates		11	2	6	2	

CD: Crohn’s disease; UC: ulcerative colitis; A1: 0-16yrs, A2: 16-40yrs, A3: >40 yrs. Location of disease in and disease behavior of CD was defined as maximal disease before surgical resection; L1: solely ileal disease, L2: solely colonic disease, L3: ileal and colonic disease, L3 + L4: ileal, colonic and upper gastrointestinal tract disease, L4: upper gastrointestinal tract disease; B1: non-stricturing, non-penetrating, B2: stricturing, B3: penetrating, (Xp): number of patients when concomitant perianal disease was present; E1: ulcerative proctitis, E2: left-sided UC, E3: pancolitis.

### RNA extraction and real-time quantitative PCR

Total RNA was extracted from the colonic mucosal biopsies using the RNeasy Mini Kit (Qiagen, Westburg BV, Leusden, The Netherlands) and converted to cDNA by reverse transcription (iScriptTM cDNA synthese kit, Biorad, CA, USA), according to the manual instructions. Real-time quantification was performed using SensiMixTM SYBR No-ROX kit (Bioline, Gentaur Europe BVBA, Kampenhout, Belgium) and 250 nM forward and reverse primers (BioLegio, Nijmegen, The Netherlands). A twostep program was run on a LightCycler® 480 II (Roche, Basel, Switzerland). Cycling conditions were 95°C for 10 minutes, 45 cycles of 95°C for 10 seconds and 60°C for 1 minute. All reactions were run in duplicate and normalized to the stably-expressed human succinate dehydrogenase complex subunit (SDHA) levels. The mRNA expression levels of the inflammatory cytokines interleukin 8 (IL-8) and tumour necrosis factor alpha (TNF-α) were analyzed as markers of inflammation. Sequences of the qRT-PCR primers and the PCR efficiencies are given in Table 2.

**Table 2:** **Sequences of used qRT**-**PCR primers and PCR efficiencies**

***Gene Symbol***	***Forward Primers*** ***(5’***-***3’)***	***Reverse Primers (5’***-***3’)***	***PCR efficiencies *** **(%)**
hSDHA	TGGGAACAAGAGGGCATCTG	CCACCACTGCATCAAATTCATG	92
hPHD1	CCGGAGGAAAAAGCTCGCCACCC	CCTCTGCGGTCCCTAAGGGCTT	105
hPHD2	CAGCATGGACGACCTGATAC	TACATAACCCGTTCCATTGC	103
hPHD3	AAAGGCGCCCTCCGACTCCT	CGACCCGTTTCCGGACTGGC	103
hIL-8	TGTTCCACTGTGCCTTGGTTTC	TGTGAGGTAAGATGGTGGCTAATAC	102
hTNF-α	ATGAGCACTGAAAGCATGATCC	GAGGGCTGATTAGAGAGAGGTC	112
hCasp3	GAGTGCTCGCAGCTCATACCT	CCTCACGGCCTGGGATTT	87

hSDHA, human succinate dehydrogenase complex subunit A; hPHD1, human prolyl hydroxylase domain 1; hPHD2, human prolyl hydroxylase domain 2; hPHD3, human prolyl hydroxylase domain 3; hIL-8, human interleukin 8; hTNF-α, human tumor necrosis factor alpha; hCasp3, human caspase 3.

### Immunohistochemistry

Paraffin-embedded colonic sections of 5 controls, 5 active UC, 5 active CD and 5 infectious colitis patients were deparaffinized with xylene, and rehydrated in a graded series of ethanol. Antigen retrieval was performed by boiling the slides in 10mM sodium citrate buffer with 0.05% Tween 20 for 20 minutes. Next, endogenous peroxidase activity was blocked with peroxidase block solution Envision (Dako) for 15 minutes. Sections were subsequently blocked with 10% goat serum for 1,5 hours at room temperature and then incubated overnight with primary antibodies at 4°C. Primairy antibodies used were rabbit monoclonal anti-PHD1 (1/50), anti-PHD2 (1/100) and rabbit polyclonal anti-PHD3 (1/200), obtained from Abcam. The slides were then treated with HRP labeled goat anti-rabbit antibody (Envision + System-HRP kit, Dako) and developed with diaminobenzidine. Counterstaining was performed with hematoxylin.

### Western blotting

Human biopsies were lysated, proteins were separated on 4-12% Bis-Tris SDS-polyacrylamide gels and transferred to nitrocellulose membranes using iBlot dry blotting (Invitrogen). Afterwards, membranes were blocked with 5% skimmed milk in TBS-T (50mM Tris (pH 7.6), 150 mM NaCl, 0.1% Tween 20) and incubated overnight at 4°C in 5% skimmed milk in TBS-T with anti-PHD1 (1/5000, Abcam), anti-PHD2 (1/600, Abcam), anti-PHD3 (1/900, Abcam) and anti-GAPDH (1/2500, Abcam). Bound antibodies were visualized using the ECL detection kit BM chemiluminescence Blotting Substrate POD (Roche) according to manufacturer’s instructions. Quantitative densitometric analysis using the Image J program was performed to quantify protein expression levels in each sample. Data were normalized to the protein expression of GAPDH.

### Statistical analysis

The data were statistically analyzed using SPSS Statistics, version 20, for Windows (SPSS, Chicago, IL). Normality of the data was checked using the Kolmogorov-Smirnoff (KS) test. In the case of normally distributed data, the differences between groups were analyzed using an unpaired Student’s t-test for independent samples. For non-normal or unknown data distribution, groups were compared by using the non-parametric Mann-Whitney U-test. The KS-test also determined the use of either a parametric (Pearson) or a non-parametric (Spearman) correlation test. Two-tailed probabilities were calculated and p-values less than or equal to 0.05 were considered statistically significant.

## Results

As a first step, we evaluated the expression of the pro-inflammatory cytokines IL-8 and TNF-alpha to confirm and define the degree of inflammation in the inflamed biopsies of IBD patients and patients with infectious colitis. It has been previously reported that they are representative markers of active inflammation [5,6]. Furthermore, we determined the expression of the apoptosis marker caspase 3 to be able to evaluate its correlation with the different PHD isoforms. The inflamed samples were characterized by highly increased IL-8, TNF-α and caspase 3 mRNA levels compared to biopsies obtained from non-inflamed areas and HC (p < 0.0001 for all groups). IL-8, TNF-α and caspase 3 expression levels in UC and CD patients in remission were comparable to those observed in the HC group (data not shown).

**Figure 1:**
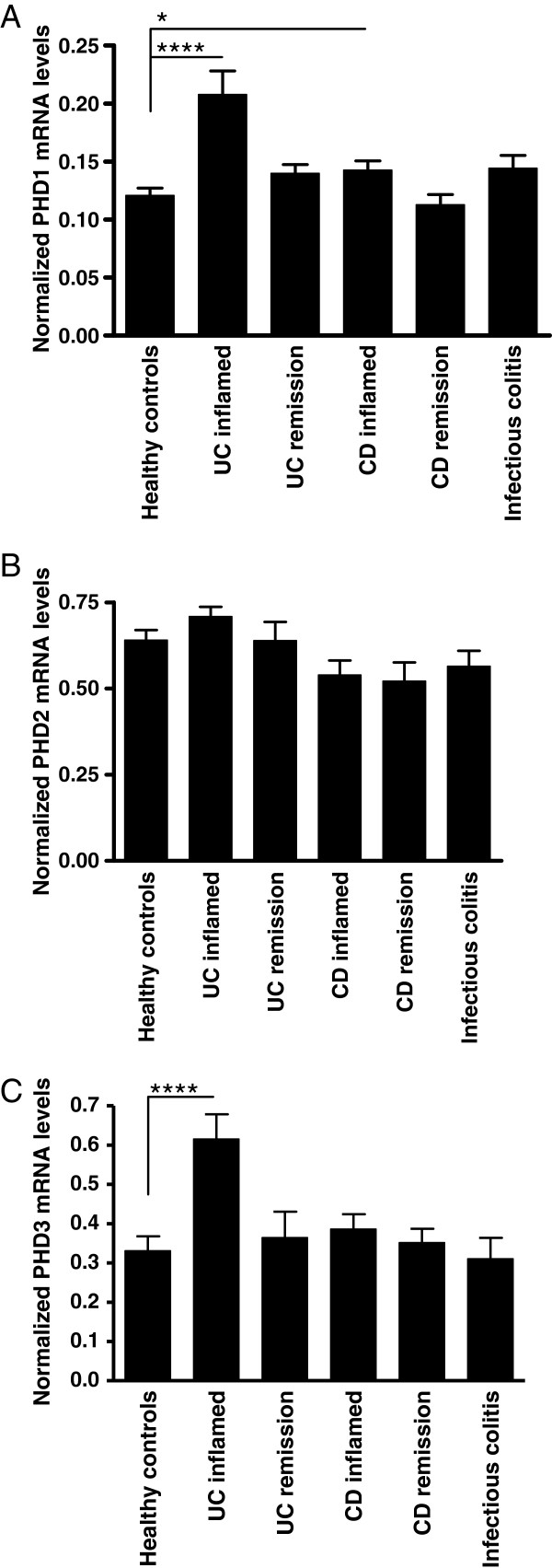
**mRNA expression of PHD1, PHD2 and PHD3 in human colonic biopsies.** mRNA expression levels of PHD1 **(A)**, PHD2 **(B)** and PHD3 **(C)** in colonic samples of healthy controls, IBD patients and patients with infectious colitis of the first patient cohort. The data are expressed as medians and presented on a log scale (****P < 0.0001, *P < 0.05).

Quantitative assessment of PHD1 mRNA levels revealed a significant increase of PHD1 in inflamed colonic biopsies of UC patients (p < 0.0001). This up-regulation was absent in patients in remission. Expression levels of PHD1 in biopsies from patients with CD and infectious colitis were only slightly elevated (p < 0.05 and p = 0.063, respectively) compared to HC, despite similarly elevated IL-8 levels (Figure 1A).

For PHD2, no differences were seen in inflamed biopsies from patients with UC, CD and infectious colitis versus non-inflamed biopsies from IBD patients in remission or healthy controls (Figure 1B).

The expression level of the PHD3 gene was significantly elevated in samples taken from inflamed colonic areas in UC patients compared to samples from HC (p < 0.0001). Inflamed samples from CD patients or infectious colitis nor non-inflamed biopsies from UC patients in remission showed an up-regulated PHD3 expression (Figure 1C).

A positive correlation was found between IL-8/TNF-α and PHD1 expression. In contrast, no correlation was found between IL-8/TNF-α and PHD2, and only a poor correlation was observed between IL-8/TNF-α and PHD3. PHD1 and, to a lesser extent, PHD2 correlated positively with caspase 3 (p < 0.0001 and p = 0.001, respectively) (Table 3).

**Table 3:** Correlations

	**PHD1**	**PHD2**	**PHD3**
**IL**-**8**	0.576 (P < 0.001)	0.089 (NS)	0.291 (P = 0.009)
**TNF**-**α**	0.706 (P < 0.001)	0.177 (NS)	0.280 (P = 0.012)
**Caspase 3**	0.594 (P < 0.0001)	0.463 (P = 0.001)	0.262 (NS)

Correlation between the expression of the inflammatory cytokines IL-8 and TNF-α, the apoptosis marker caspase 3 and the expression of the different PHD isoforms in colonic mucosal biopsies (Pearsons r for IL-8 and TNF-α, Spearman’s r for caspase 3).

All above reported results were confirmed in a second, independent patient cohort (data not shown).

**Figure 2:**
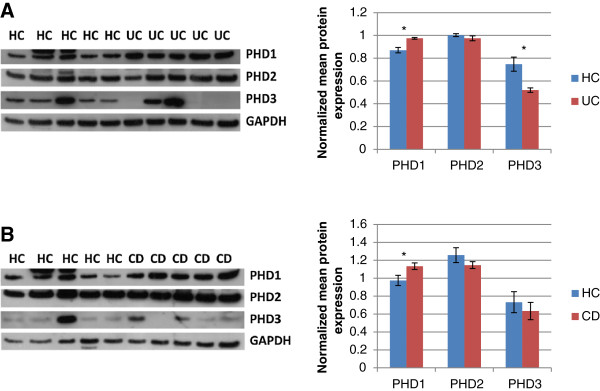
**Protein expression levels of PHD1**, **2 and 3 in human colonic samples. A)** Protein expression of PHD1, PHD2 and PHD3 in whole biopsy lysates of 5 healthy controls and 5 UC patients (lane 6, 9 and 10: severe UC and lane 7 and 8:mild to moderate UC). **B)** Protein expression of PHD1, PHD2 and PHD3 in whole biopsy lysates of 5 healthy controls and 5 CD patients (lane 7 and 8: severe CD and lane 6, 9 and 10: mild to moderate CD). The columns represent the densitometric evaluation of the PHDs, normalized to GAPDH (mean ± SEM)(*P < 0.05).

Next, the protein expression levels of the three PHD isoforms were evaluated in biopsies of 5 healthy controls and in inflamed biopsies of 5 UC patients (3 with severe disease (Mayo endoscopic score 3), 2 with mild to moderate disease (Mayo endoscopic score 1-2)) and 5 CD patients (2 with severe disease and 3 with mild to moderate disease). As shown in Figure 2A and Figure 2B, PHD1 protein expression was significantly increased in both UC (p < 0.05) and CD (P < 0.05) patients compared to healthy controls. PHD2 protein levels were not altered between all groups. The PHD3 protein expression was not significantly different between inflamed samples of CD patients versus healthy controls (Figure 2B). However, the expression in the inflamed samples from severely diseased UC patients (Figure 2A; lane 6, 9 and 10) was significantly lower compared to healthy controls,.

**Figure 3:**
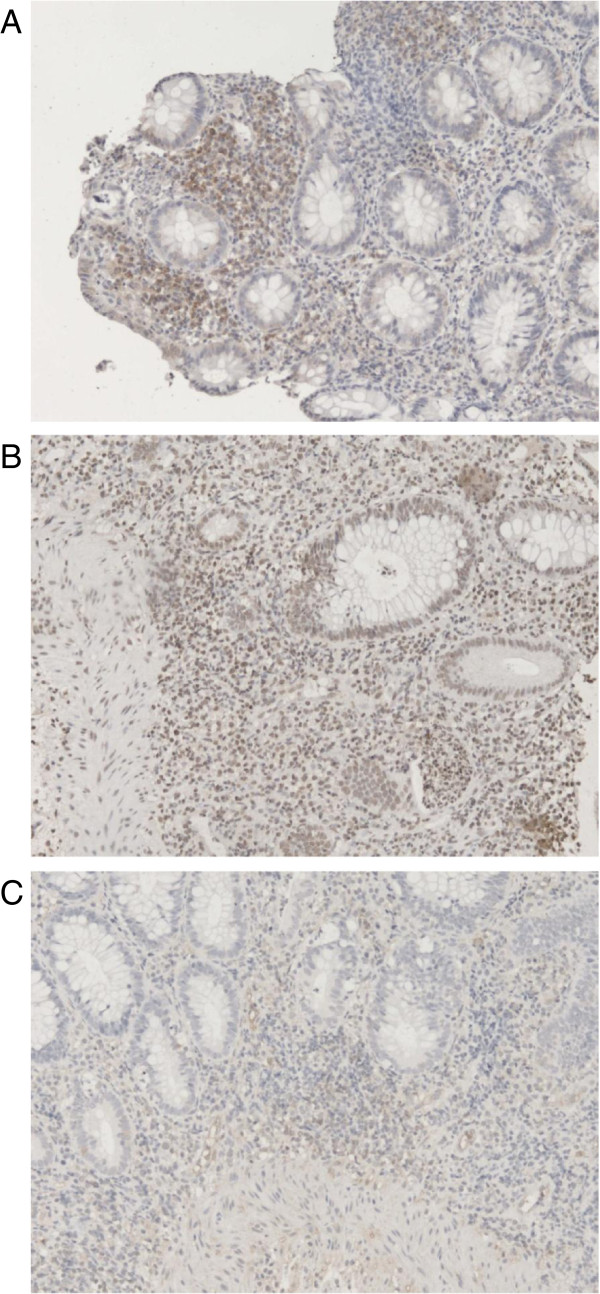
**Immunostaining of human biopsies for PHD1**, **PHD2 and PHD3. A)** Immunostaining of PHD1 demonstrates cytoplasmatic staining of mononuclear cells in the lamina propria and of regenerative epithelium. **B)** Immunostaining of PHD2 shows nuclear staining of epithelium, mononuclear cells in the lamina propria and smooth muscle cells in the muscularis mucosae. **C)** Immunostaining of PHD3 reveals selective staining of the endothelium of blood vessels. Only the representative images (200x) of UC patients are given as no disease-dependent localization of the PHDs was observed.

On immunohistochemistry, no disease-dependant localization of the PHDs was observed. PHD1 was predominantly found in regenerative epithelial cells and in the cytoplasm of mononuclear cells (e.g. dendritic cells, macrophages) in the lamina propria (Figure 3A). Lymphocytes were PHD1 negative. For PHD2, we observed strong nuclear staining in a wider range of cell types than for PHD1. Approximately half of the cells in the epithelium, inflammatory cell infiltrate (mononuclear cells in the lamina propria and lymphocytes) and smooth muscle cells in the muscularis mucosae showed strong PHD2 staining (Figure 3B). Lastly, we found that the PHD3 protein is specifically located in the endothelium of blood vessels (Figure 3C).

## Discussion

In this study, we analyzed the expression and the localization of the different PHD isoforms in IBD patients, in order to identify the primary target(s) for the development of specific PHD-inhibitors.

The current treatment strategy for both CD and UC is focused on the suppression of inflammation. Standard therapy includes corticosteroids, 5-ASA preparations, immunomodulating drugs and/or biologicals. Despite these drugs, approximately 70% of the patients with CD and 35% of patients with UC ultimately come to surgery. Therefore, research in IBD is still focused on the identification of novel therapeutics to improve the disease outcome. In this regard, panhydroxylase inhibitors have been proposed as promising therapeutic compounds for IBD [1-4], but most of these studies lack human data to support their claim.

We found strongly increased mRNA expression of PHD1 and PHD3 in inflamed biopsies from patients with UC whereas inflamed biopsies from patients with CD and infectious colitis only displayed a slight increase in PHD1 expression. Only PHD1 showed a good correlation with the pro-inflammatory markers IL-8 and TNF-α. Whether or not inflammatory cytokines directly influence the PHD expression or vice versa is a subject of further research.

In accordance with our mRNA results, a significant elevation of PHD1 protein expression was observed in inflamed biopsies of both UC and CD patients (p < 0.05) while PHD2 protein levels remained unaltered. PHD3 protein expressions were comparable between all groups except for UC patients. In contrast to the mRNA levels, severely diseased UC patients displayed a significant decrease in PHD3 expression (p < 0.05). This might, at least in part, be explained by the fact that Siah 2, a E3 ubiquitine ligase, becomes activated as oxygen concentration decreases due to the extensive consumption of oxygen by the inflammatory cells, leading to the proteasomal degradation of PHD3 [7,8]. The same phenomenon was not seen in CD patients, where PHD3 expression did not follow the severity of the disease. This is not unexpected because biopsies from CD patients are always characterized by a discontinuous infiltrate of inflammatory cells so that the fluctuating levels of high and low oxygen give rise to a net hypoxic situation that is less pronounced than in patients with severe UC.

Apart from a role in inflammation, a role of PHDs in apoptosis has also been suggested. It has been shown that inhibition of PHD1 and PHD2 results in activation of HIF-1α and NF-κB [9], both being transcription factors that regulate the expression of several genes involved in apoptosis [10]. Inflammatory bowel disease is hallmarked by an increased rate of intestinal epithelial cell death. In fact, one of the main mechanisms of action by which panhydroxylase inhibitors are able to suppress experimental colitis, is probably by reducing colonic epithelial cell apoptosis [11]. Our data also indirectly imply a role of PHD1 and PHD2 in colonic epithelial apoptosis as these isoforms show a positive correlation with caspase 3, a marker of apoptosis. It are exactly these isoforms that can be found in the colonic epithelium.

In conclusion, only PHD1 was up-regulated both at the mRNA and the protein level and showed an excellent correlation with both inflammatory markers and apoptosis in IBD (especially in UC). Allthough we acknowledge that PHD1 protein expression as such is not directly related to its enzymatic activity, our exploratory expression analysis puts PHD1 forward as the primary therapeutic target for UC and, to a lesser extent, for colonic CD. This is further supported by the observation that PHD1-deficient mice, and not PHD2- or PHD3-deficient ones, are highly protected against colitis by reducing epithelial cell apoptosis and hence, by maintaining barrier function [12].

## Abbreviations

PHD: Prolyl hydroxylase domain-containing protein; IBD: Inflammatory bowel disease; CD: Crohn's disease; UC: Ulcerative colitis.

## Competing interests

None of the authors have competing interest with regard to the manuscript.

## Authors' contributions

SVW carried out the studies and data analyses and drafted the manuscript. DL supervised samples analyses and statistics. MDV participated in the design and coordination of the study. PH conceived of the study, and participated in its design and coordination and helped to draft the manuscript. All authors read and approved the final manuscript.
